# DNA Methylation of T Lymphocytes as a Therapeutic Target: Implications for Rheumatoid Arthritis Etiology

**DOI:** 10.3389/fimmu.2022.863703

**Published:** 2022-03-03

**Authors:** Jianan Zhao, Kai Wei, Cen Chang, Lingxia Xu, Ping Jiang, Shicheng Guo, Steven J. Schrodi, Dongyi He

**Affiliations:** ^1^ Guanghua Clinical Medical College, Shanghai University of Traditional Chinese Medicine, Shanghai, China; ^2^ Department of Rheumatology, Shanghai Guanghua Hospital, Shanghai University of Traditional Chinese Medicine, Shanghai, China; ^3^ Institute of Arthritis Research in Integrative Medicine, Shanghai Academy of Traditional Chinese Medicine, Shanghai, China; ^4^ Computation and Informatics in Biology and Medicine, University of Wisconsin-Madison, Madison, WI, United States; ^5^ Department of Medical Genetics, School of Medicine and Public Health, University of Wisconsin-Madison, Madison, WI, United States; ^6^ Arthritis Institute of Integrated Traditional and Western medicine, Shanghai Chinese Medicine Research Institute, Shanghai, China

**Keywords:** rheumatoid arthritis, DNA methylation, T-cell, precision medicine, therapeutic target

## Abstract

Rheumatoid arthritis (RA) is an autoimmune disease that can cause joint damage and disability. Epigenetic variation, especially DNA methylation, has been shown to be involved in almost all the stages of the pathology of RA, from autoantibody production to various self-effector T cells and the defects of protective T cells that can lead to chronic inflammation and erosion of bones and joints. Given the critical role of T cells in the pathology of RA, the regulatory functions of DNA methylation in T cell biology remain unclear. In this review, we elaborate on the relationship between RA pathogenesis and DNA methylation in the context of different T cell populations. We summarize the relevant methylation events in T cell development, differentiation, and T cell-related genes in disease prediction and drug efficacy. Understanding the epigenetic regulation of T cells has the potential to profoundly translate preclinical results into clinical practice and provide a framework for the development of novel, individualized RA therapeutics.

## Introduction

Rheumatoid arthritis (RA) is a chronic and systemic inflammatory illness that causes persistent synovial inflammation and bone and joint damage. RA increases the risk of various diseases such as respiratory infections, osteoporosis, cardiovascular disease, urinary tract disease, and cancer (1). The incidence in women is higher than that in men. RA has a somewhat bimodal distribution of age of onset, with the largest fraction initially being diagnosed in the 20-40s, and then a smaller fraction being diagnosed roughly in the 60s, and it can appear at any stage (1). Typically, numerous autoimmune antibodies and cytokines are present in the pre-onset stage, and this molecular signature further advances with the clinical onset of synovial hyperplasia and bone/joint damage. In the absence of effective therapy, the disease typically progresses, resulting in impairment of both physical and mental health (2).

**Figure 1:**
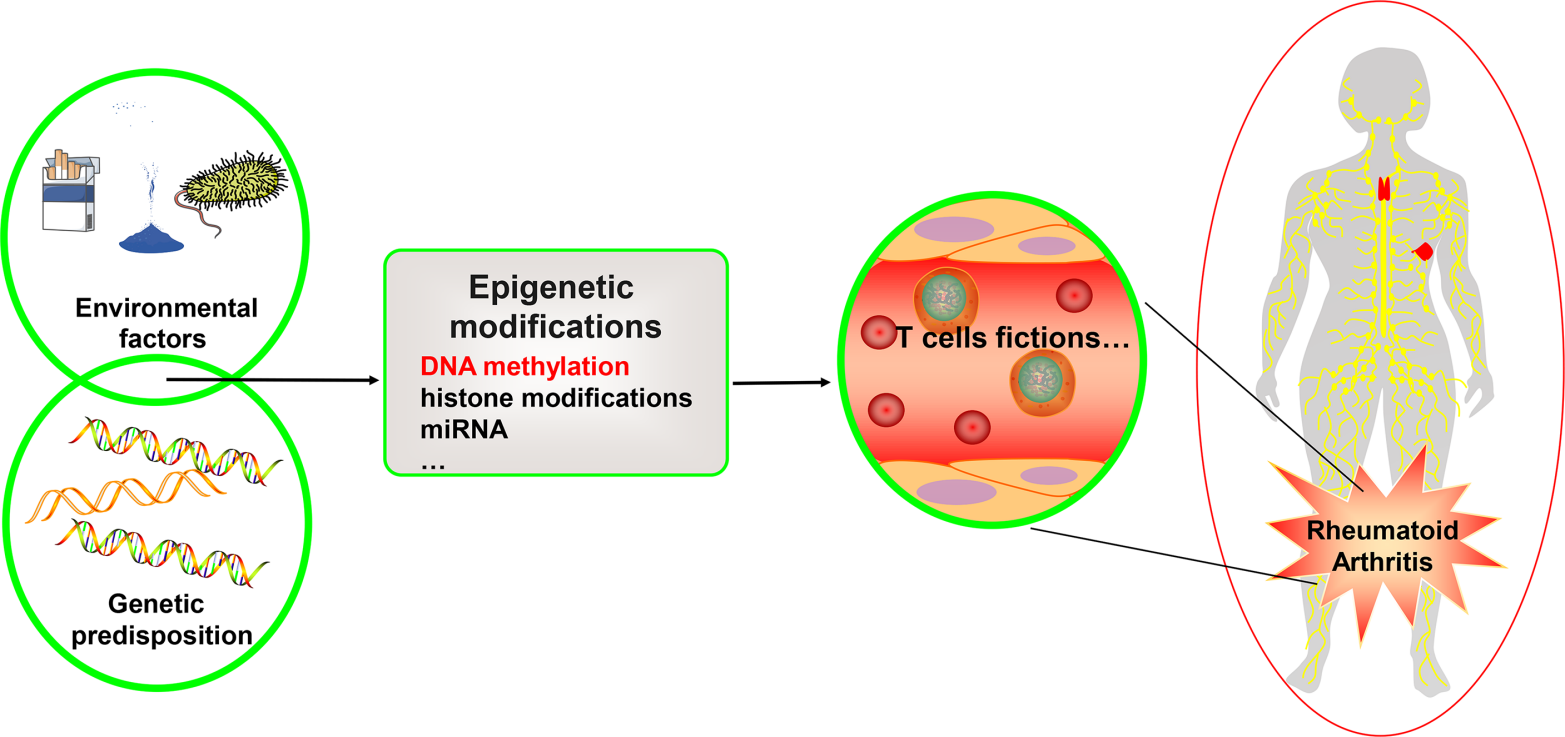
Genetics, environment, and DNA methylation are interrelated in T cell function for rheumatoid arthritis. Rheumatoid arthritis is a heritable autoimmune disease. Some gene variants can increase risk of disease. Environmental factors interact with the genetic background and can affect the function of the T cell subset, thus affecting rheumatoid arthritis through different epigenetic mechanisms, including DNA methylation, histone modifications, and microRNAs. We focus on the DNA methylation landscape in T cells.

Many factors influence RA, including heredity, metabolism, environmental factors, and microbiota– and involve a complex interplay of genetic predisposition, activation of a variety of immune cells, and imbalance of pro- and anti-inflammatory mechanisms. This pathobiological immune state ultimately drives the failure to regulate immune tolerance, causing the development of autoimmune diseases targeting the synovium (3–5) (see **Figure 1**). Current research has discovered that numerous gene variants contribute to illness development, implying that each susceptible gene variant contributes to the disease to varying degrees (6). In addition to gene variations, the interaction between the environment and genes influences gene expression, which is heavily influenced by epigenetic modifications. This includes DNA methylation, histone post-translational modification, miRNA, and, eventually, immune system regulation (6). DNA methylation refers to the addition of a methyl group to cytosine guanine (CG) dinucleotide (CpG). Two-thirds of CpGs in the human genome can be methylated (7). Under normal conditions, the 5-methylcytosine of CpG islands in DNA methylation prevents transcription factor complexes from binding to DNA through multiple processes, resulting in the inhibition of gene expression. On the other hand, DNA hypermethylation of gene promoters can enhance downstream gene transcription through a variety of mechanisms. Promoter hypermethylation, for example, blocks transcription inhibitors from binding to DNA (8). Hypermethylated advocates engage with enhancers to attract transcriptional activators and improve downstream gene transcription. Hypermethylation also promotes gene expression by binding to enhancers instead of insulators. Furthermore, in mammals, the hypermethylated promoter stimulates transcription of the first promoter of multiple variable promoters, resulting in increased target gene transcript output and decreased alternative spliceosome content (8).

T cells primarily engage with antigen-presenting cells and mix with antigen peptides provided by human leukocyte antigen (HLA) to create complexes that further mediate immunological responses (9). DNA methylation can influence various degrees of gene expression or selective splicing, both of which are associated with RA (10). The biological roles of the majority of the differential genes were shown to be associated with T cell development and activation by assessing gene expression and DNA methylation of CD4+ T cells in RA patients and healthy individuals (11). Many differentially methylated sites are found in T cell regulatory regions. Most gene variants were moderately or strongly correlated with methylation quantitative trait loci (meQTLs). Differentially variable positions (DVPs) are a type of CpG site. By studying the methylation of whole-genome DNA in monozygotic twins with discordant conditions, it was found that 1171 RA-related DVPs, of which 763 DVPs are in a highly variable state in RA twins, were mainly enriched in gene bodies and the 3’UTR of genes. Of the 763 DVPs, 563 were hypomethylated (12). Pathway analysis revealed that two of the top five were linked to T cells and cytokines (12). This suggests that the differential expression of RA genes may be partly driven by DNA methylation and are linked to variants of disease susceptibility genes that contribute to disease progression (11). In addition, there are several aberrant T-cell patterns in RA patients. In RA patients, both CD4+ naïve T cells and memory T cells exhibit premature aging and excessive telomere degradation (13). Furthermore, the ataxia telangiectasia mutated defect of T cells in RA patients leads to the destruction of the DNA repair mechanism (14). It enhances the sensitivity of T cells to apoptosis, while the apoptosis attrition of naive T cells ultimately accelerates immune senescence and the proliferation of autoimmune T cells (14). DNA methylation may cause aberrant activation and premature aging of T cells, contributing to atypical T-cell patterns in RA. In this article, we summarize and discuss the relationship between T cell DNA methylation and the pathological mechanism of RA in order to provide a theoretical foundation and reference for the development of novel clinical diagnosis and treatment programs from an epigenetic perspective (See **Table 1**).

**Table 1:** Relationship between T cell DNA methylation-related genes and rheumatoid arthritis.

Gene	States in RA	Potential association with RA	Ref.
TYK2	hypermethylation	It is related to the susceptibility of RA	(15)
PRKAR1B	hypermethylation	It is related to the susceptibility of RA	(15)
ABCC4	hypermethylation	The genetic variation of ABCC4 is related to the efficacy of Ginsenoside compound K in the treatment of RA.	(15, 16)
COMT	hypermethylation	COMT is an enzyme that degrades catecholamines. Catecholamines can regulate cell proliferation and differentiation, apoptosis, and cytokine production. Abnormal metabolism of catecholamines in immune cells is related to RA.	(15, 17)
MCF2L	hypermethylation	It is related to the susceptibility of RA	(15)
GALNT9	hypermethylation	It is related to the susceptibility of RA	(15)
C7orf50	hypermethylation	It is related to the susceptibility of RA	(15)
HDAC4	hypermethylation	The activation of HDAC4 can inhibit the proliferation, migration and inflammation of RA FLS.	(18, 19)
NXN	hypermethylation	CD+ T cell DNA differential methylation genes in Asian population	(18)
TBCD	hypermethylation	CD+ T cell DNA differential methylation genes in Asian population	(18)
TMEM61	hypermethylation	CD+ T cell DNA differential methylation genes in Asian population	(18)
JUN	hypermethylation	It regulates cell proliferation, cell cycle and apoptosis	(20)
STAT1	hypermethylation	It is related to IL-6 inflammatory factor	(21)
PTEN	hypermethylation	It is related to cell proliferation and production of pro-inflammatory factors.	(22, 23)
CD44	hypermethylation	It is a cell adhesion factor, which can increase the incidence	(24)
T-bet	hypermethylation	It is related to Th1 differentiation	(25)
Foxp3	hypermethylation	Development and functional integrity of Treg, pharmacological mechanism of MTX	(26, 27)
CTLA4	hypermethylation	It is related to the inhibitory function of Treg and related to the pharmacological mechanism of MTX	(27)
C6orf47	hypermethylation	DNA methylation changes in early RA patients	(28)
AGPAT9	hypermethylation	DNA methylation changes in early RA patients	(28)
DUSP22	hypermethylation	Hypomethylated regions in the DUSP22 gene promoters were associated with active and erosive disease.	(28)
SLC25A13	hypermethylation	DNA methylation changes in early RA patients	(28)
SNORA15	hypermethylation	DNA methylation changes in early RA patients	(28)
C1orf106	hypermethylation	Changes in DNA methylation in RA patients	(29)
ZFP57	hypermethylation	Changes in DNA methylation in RA patients	(29)
ARSB	hypermethylation	It is related to the formation and function of lysosomes.	(29, 30)
LSM5	hypermethylation	Changes in DNA methylation in RA patients	(29)
HIST1H4D	hypermethylation	Changes in DNA methylation in RA patients	(29)
OR5A2	hypermethylation	It is related to the course of RA	(18)
C5orf32	hypermethylation	It is related to the course of RA	(18)
C16orf71	hypermethylation	It is related to disease activity score in 28 Joints	(18)
C18orf19	hypermethylation	It is related to erythrocyte sedimentation rate	(18)
COL18A1	hypermethylation	It is related to erythrocyte sedimentation rate	(18)
BAT3	hypermethylation	It is related to erythrocyte sedimentation rate	(18)
PLD3	hypermethylation	It is related to erythrocyte sedimentation rate	(18)
HSPA12A	hypermethylation	It is related to tender joint count	(18)
SMAD7	hypermethylation	It increases a variety of immune inflammatory responses	(31, 32)
CD83	hypermethylation	It is a key differentiation factor for Treg cells	(31, 32)
HLA-DRB6	hypomethylation	It is associated with increased risk for RA	(18, 33)
HLA-DQA1	hypomethylation	It is associated with an increased rate of immunogenicity	(18, 34)
HLA-E	hypomethylation	Its polymorphisms may influence RA susceptibility and affect clinical outcome of anti-TNF therapy in female RA patients	(18, 35)
ITIH3	hypomethylation	Citrullinated ITIH3 as a potential diagnostic and pathological marker of RA	(18, 36)
TCN2	hypomethylation	CD+ T cell DNA differential methylation genes in Asian population	(18)
PRDM16	hypomethylation	CD+ T cell DNA differential methylation genes in Asian population	(18)
SLC1A5	hypomethylation	It belongs to the gene that regulates cell metabolism and potentially regulates the inflammation of RA.	(18, 37)
GALNT9	hypomethylation	CD+ T cell DNA differential methylation genes in Asian population	(18)
KRAS	hypomethylation	It lowers the activation threshold of T cells	(38)
IFNG	hypomethylation	It helps Th1 cells to differentiate	(39)
DNMT3a	hypomethylation	It affects T cell differentiation	(40)
UBASH3A	hypomethylation	It is related to antigen presentation to T cells	(41, 42)
CD40L	hypomethylation	It is associated with an increase in the prevalence of women with RA and it can inhibit CD4+T cell.	(43, 44)
IL-32	hypomethylation	It is associated with chronic inflammation	(45)
MRPL28	hypomethylation	It is related to mitochondrial function	(46, 47)
ALB	hypomethylation	It is related to the pharmacological mechanism of MTX	(27)
GATA3	hypomethylation	It is related to the pharmacological mechanism of MTX	(48, 49)
GATA3-AS1	hypomethylation	It is related to the pharmacological mechanism of MTX	(48, 49)
IL-6	hypomethylation	It is related to the pharmacological mechanism of IL-6R therapy	(50)
IL-6R	hypomethylation	It is related to the pharmacological mechanism of IL-6R therapy	(50)
MAGI2	hypomethylation	DNA methylation changes in early RA patients	(28)
C11orf58	hypomethylation	DNA methylation changes in early RA patients	(28)
POMZP3	hypomethylation	DNA methylation changes in early RA patients	(28)
ZNF708	hypomethylation	DNA methylation changes in early RA patients	(28)
CLEC2L	hypomethylation	DNA methylation changes in early RA patients	(28)
TMEM9	hypomethylation	Changes in DNA methylation in RA patients	(29)
CCS	hypomethylation	Changes in DNA methylation in RA patients	(29)
FBRSL1	hypomethylation	Changes in DNA methylation in RA patients	(29)
GALNT9	hypomethylation	Changes in DNA methylation in RA patients	(29)
PDHB	hypomethylation	Changes in DNA methylation in RA patients	(29)
MGMT	hypomethylation	It may be related to the DNA repair mechanism	(29, 51)
DDO	hypomethylation	Changes in DNA methylation in RA patients	(29)
KLF6	hypomethylation	Changes in DNA methylation in RA patients	(29)
RDH12	hypomethylation	Changes in DNA methylation in RA patients	(29)
ALDH9A1	hypomethylation	It is related to the course of RA	(18)
ZC3H11A	hypomethylation	It is related to RF	(18)
OAS2	hypomethylation	It is related to patient global assessment	(18)
LOC100129716	hypomethylation	It is related to disease activity score in 28 Joints	(18)
SLC38A8	hypomethylation	It is related to erythrocyte sedimentation rate	(18)
CAI2	*NA*	It is related to the susceptibility of RA	(15)
RPH3AL	*NA*	It is related to the pharmacological mechanism of MTX	(52)
WDR27	*NA*	It is related to the pharmacological mechanism of MTX and TNF-a inhibitors	(52, 53)

^*^NA, not available.

## The Potential Connection Between DNA Methylation of CD4+ T Cells and Rheumatoid Arthritis

Several intriguing results were discovered using transcriptomic data, methylation data, and bioinformatics to analyze the differential genes and differentially methylated genes of diverse immune cells in RA (54). First, the presence of anti-lymphocyte antibodies may have resulted in a considerable reduction in the total number of T and B cells in the peripheral blood of patients with RA compared to healthy controls (54, 55). Second, RA patient CD4+ T cells exhibit hypermethylated genes, including *JUN, STAT1, PTEN*, and *CD44*, compared to healthy controls, whereas hypomethylated genes include *KRAS* and *ALB*. Additionally, the expression levels of *STAT5B, SOCS3, JUN, STAT1, KRAS, ALB*, and *CD44* were increased in RA. *PTEN, FGFR2*, and *DICER1* expression levels were reduced. Gene ontology (GO) enrichment studies of differentially methylated genes are connected to T cell biological processes, suggesting that the regulation of CD4+ cells *via* DNA methylation plays an important role in RA (54). Finally, the analysis of the differentially methylated genes in the Kyoto Encyclopedia of Genes and Genomes (KEGG) showed that it was mainly related to thyroid hormone and estrogen signaling, hepatitis B virus (HBV), and the mitogen-activated protein kinase (MAPK) pathway (54). The results of the KEGG pathway analysis suggested various potential connections between gene DNA methylation and RA. Patients with RA have a higher prevalence of thyroid disease (56, 57). It often shows a certain degree of hypothyroidism at onset, which is used as a sign of autoimmune thyroiditis (58). RA and HBV are interconnected and have been widely studied (59). For example, RA patients receiving tofacitinib can induce HBV reactivation in HBsAg+ and HBsAg-/HBcAb+ patients (60), and HBV exposure correlates with rheumatoid factor (RF) (61). The increased incidence of RA in postmenopausal women may be related to the lack of estrogen. Studies have found that estrogen can promote the degradation of acid-sensitive ion channel 1a protein and inhibit the cytotoxicity of acidosis through autophagy-lysosome-dependent pathways, thereby protecting chondrocytes (62, 63).

By comparing the number of differentially methylated positions of memory CD4+ T cells and CD4+ naive T cells in RA patients, it was found that the number of the former was significantly greater than the latter. Most of the DNA methylation of differentially methylated positions (DMP) in RA patients with active disease is increased (64). Among the 101 RA genes recently identified (65), more than 30 overlapped with DMP (*P*=0.1 after 1000 permutations). One of these, *UBASH3A*, was found in CD4+ memory T cells. Its introns contain both single nucleotide polymorphisms (SNPs) that are correlated with RA risk r and two DMP and show a decrease in DNA methylation, which may increase gene expression (64). The protein encoded by *UBASH3A*, ubiquitin-associated and SH3 domain-containing A protein, primarily plays a role in the presentation of antigens to T cells and dampens T cell activity (41, 42). Therefore, the expression of *UBASH3A* may increase the expression of genes through DNA methylation to promote antigen presentation to autoimmune T cells. *UBASH3A* SNPs (rs1893592, rs3788013), rheumatoid factor, cyclic citrullinated peptide (anti-CCP), disease activity score in 28 joints (DAS28), and C-reactive protein (CRP) were all significantly correlated with RA susceptibility (66, 67). By studying peripheral blood samples from RA patients, researchers have found that CD4+ T memory cells have one hypermethylated CpG site, and CD4+ naïve T cells have 18 CpG sites; six are hypomethylated, and 12 are hypermethylated. The most important top 10 sites were located in *TYK2, PRKAR1B, ABCC4, COMT, CAI2, MCF2L, GALNT9, C7orf50*, and two intergenic regions (15). All 19 CpG sites were related to RA susceptibility. The hypomethylation status of CpG sites in the promoter regions of *GATA3* and *GATA3-AS1* (cg17566118 and cg15852223) was also found (15). The meQTL-mediated differentially methylated regions lead to the downregulation of *GATA3* and *CD83* expression (11). *GATA3* is a transcription factor that drives the differentiation of anti-inflammatory Th2 cells, promotes the enhancement of the interleukin (IL)-4 promoter, and regulates the expression of the IL-4 gene. The decrease in its expression may further lead to an imbalance in anti-inflammatory mechanisms (48, 49). Oral administration of a-L-Guluronic acid, a non-steroidal anti-inflammatory drug (NSAID), can significantly increase the expression of *IL-4* and *GATA3* in peripheral blood mononuclear cells (PBMCs) and improve the symptoms of related diseases in RA patients (68). CD4+ T cells lacking *CD83* exhibit enhanced pro-inflammatory differentiation ability under *in vitro* stimulation; for example, more interferon-gamma (IFN-γ) secretion and more Th1 and Th17 cell differentiation (31). The study detected genome-wide DNA methylation of CD4+ T cells from RA patients of Han Chinese ancestry. This reveals RA-related DNA methylation patterns in East Asian populations (18). There were 383 hypomethylation genes and 785 hypermethylated genes in CD4+ T cells. *HDAC4, NXN, TBCD, *and *TMEM61* are the top four hypermethylated genes. *HLA-DRB6, HLA-DQA1, HLA-E, ITIH3, TCN2, PRDM16, SLC1A5*, and* GALNT9 *were hypomethylated. *HLA-DQB1* is hypermethylated in the CpG island region, and the CpG framework is hypomethylated (18). In addition, the DNA methylation mechanism may also be a reason for the higher prevalence of RA in women. The hypomethylation status of *CD40L* in CD4+ T cells of female patients with RA is conducive to the expression of *CD40L* and partially explains the higher prevalence of RA in women (43). Consistent with this, the expression of 1,25(OH)_2_D_3_ and the vitamin D receptor (VDR) in the peripheral blood of RA patients is reduced. 1,25(OH)_2_D_3_ and VDR can inhibit the PKCδ/ERK pathway and promote DNA methylation of *CD11a, CD70*, and *CD40L* to inhibit CD4+ T cell activation (44).

In this section, we summarize the changes in CD4+ T cell-related differential DNA methylation, such as *JUN, PTEN*, and *STAT1*. Next, we explored the potential connection between its related genes and RA. Cell survival and death of CD4+ T cells play an essential role in RA (46). The upregulation of c-Jun expression* *can activate the MAPK signaling pathway and regulate cell proliferation, cell cycle, and apoptosis (20). Both *STAT1* and *STAT3* are related to IL-6 inflammatory factors and are involved in the course of RA (21). *Gp130/STAT1* may increase the joint infiltration of T cells in antigen-induced arthritis (AIA) animal models (69). *PTEN* is mainly involved in cell proliferation and pro-inflammatory factors. Overexpression of PTEN can significantly reduce the expression of pro-inflammatory factors, chemokines, vascular cell adhesion factor-1, and vascular endothelial growth factor-α in AIA experimental animals. Consistent with the previous, the expression of PTEN is regulated by DNA methylation and is accompanied by changes in the AKT signal (22), and the methylation of PTEN has been shown to promote inflammation and activation of RA fibroblast-like synoviocyte (FLS) (23). Shikonin significantly reduces angiogenesis in RA. The mechanism may involve up-regulating the expression of PTEN, downregulating the expression of tumor necrosis factor-a (TNF-a), vascular endothelial growth factor 2, and IL-1β, and inhibiting the phosphorylation and gene expression of *ERK1/2, JNK1/2*, and* P38* induced by TNF-α (70). In addition, *PTEN* is regulated by various miRNAs. Tocilizumab can regulate the proliferation of RA FLS and the production of pro-inflammatory factors *via* the MIR31HG/miR-214/PTEN/AKT axis (71). Therefore, *PTEN* may be a vital factor in inflammation in RA. CD44 is mainly used as a cell-surface adhesion molecule (72). The anti-CD44 antibody can significantly increase the incidence of experimental arthritis mice induced by type II collagen (24). Studies have shown that the preclinical stage of the disease in the K/BxN mouse model reduced CD4+ T lymphocytes. However, in the early clinical setting, most of the CD4+ T cells begin to express CD44, which is regarded as a sign of cell homeostasis expansion (73). Selenium supplementation can improve the joint pathological performance of CIA mouse models, reduce the number of CD4+CD44+ receptor activators of nuclear factor (NF)-κB ligand (RANKL) +T cells, and inhibit the expression of RANKL in mouse Th17 cells *in vitro* (74). Osteopontin in the synovial fluid of patients with RA induces Th17 differentiation and promotes the production of pro-inflammatory factor IL-17 through the IL-6/STAT3 pathway and CD44, CD29, and retinoic acid-related orphan receptor (ROR). The mechanism may be CD44 binding domain of CD4+ T cells induces H3 acetylation of the *IL-17A* promoter and promotes interaction with ROR (75). The overexpression of* KRAS* and *BRAF* in CD4+ T cells lowers the activation threshold of T cells to respond more efficiently to autoantigens in RA. This is related to the level of phosphorylated ERK (38).

## The Effect of DNA Methylation on the Differentiation of CD4+ T Cells

T cells have cellular heterogeneity and can differentiate into different subsets under stimulation by different cytokines, such as Treg cells, Th1 cells, Th2 cells, Th17 cells, and T-follicular helper (Tfh) cells, which play different roles in RA (76). Th1 and Th17 cell subsets contribute to inflammation in RA. Many studies have shown that the inhibition of Th1 and Th17 cell subsets can alleviate RA (77–79). Th2 cell subsets can inhibit Th1 and Th17 cell differentiation and function by secreting IL-4 as a mechanism to antagonize inflammation (80). In early patients with RA, Th2 cells and their secreted cytokines are few, and the differentiation of CD4+ T cells is severely unbalanced, which significantly promotes the production of chronic inflammation (81). Studies have shown that T cells lacking DNA methyltransferase *(DNMT) 3a* are activated and produce IL-4 and *IFNG* site hypomethylation. When T cells with Th2 cell characteristics are biased to induce Th1 cells, they will express more IFN-γ, indicating that DNA methylation plays a vital role in controlling T cell differentiation according to the microenvironment (82). Further research shows that *DNMT3a* is an essential element that controls the differentiation of Th2 cells, expresses IL-13, and maintains the function of Th2 cells to inhibit inflammation (40). There is a substantial body of literature on how epigenetic mechanisms, including DNA methylation, affect the differentiation of lymphoid T cells (26, 83).

In response to IL-12 stimulation, CD4+ T cells differentiate into Th1 cells and produce IFN-γ. When activated for the first time, intracellular *IFNG* can be expressed under the stimulus of TCR signals and can bind to the transcription factor *T-bet* or *STAT4*. IL- 12 can also reactivate the *IFNG* of memory Th1 cells for a second time (84–86). Studies have shown that the hypomethylation pattern of the *IFNG* promoter region and the CNS-1 region may contribute to the differentiation of Th1 and establish a stable epigenetic imprint, which is conducive to the reactivation of Th1 and promotes inflammation (39). Many meaningful results have been obtained by detecting the differential DNA methylation sites of monocytes, memory CD4+ T cells, and CD4+ naïve T cells in patients with RA (87). First, 1047 and 913 differentially methylated CpGs were detected in CD4+ naïve T cells and memory CD4+ T cells, respectively. Second, CD4+ T cells in early RA patients seem to develop in the direction of Th17 cells. Differential methylation of *TBX21* (*T-bet* in mice) may lead to a decrease in Th1 cell differentiation (85). At the same time, the *IL-17* and *IL-17R* of CD4+naïve T cells undergo differential methylation modification, which tend to differentiate into pro-inflammatory Th17 cells and interact with TNF. Differential methylation of IFN-related genes affects gene expression (87). As previously reported, it may indicate the characteristics of chronic inflammation and predict the progression of arthritis in early RA (88, 89). Interestingly, when T cells were treated with DNA methyltransferase inhibitors, the expression of STAT4 was significantly upregulated. The shortening of the methylation site of the proximal regulatory element of its promoter greatly enhanced transcriptional activity, indicating that STAT4 in T cells is affected by DNA methylation regulation and is not regulated by promoter polymorphism (90). Decitabine, a DNA methylation inhibitor, can significantly improve the clinical symptoms of experimental arthritis mice induced by type II collagen, inhibit pro-inflammatory cytokines of Th1 and Th17 cells, reduce the production of anti-type II collagen antibodies (91), and promote the production of cytokines by Th2 cells (92). In addition, Tfh cells mainly promote B cell antibody maturation and development (93). The binding of BCL6, an essential molecule for Tfh cell differentiation, is associated with a decrease in 5-hydroxymethylcytosine (5hmC), implying a link between Tfh and DNA methylation (94). Hence, DNA methylation can regulate the differentiation of various T cell subsets *via* different mechanisms that affect the development of patients with RA.

## The Defects of DNA Methylation in Treg Cells Promote Inflammation

Treg cells are defined as CD4+CD25+FOXP3+; their expression defects contribute to the production of autoimmunity because their functions can inhibit the proliferation and differentiation of autoimmune T cells (95). Treg cells also have cellular heterogeneity, mainly divided into resting-state CD45^+^FOXP3^low^ Treg cells, activated CD45RA^-^FOXP3^hi^, and CD45^-^ FOXP3^low^Treg cells, and a subset of Treg cells expressing *HLA-DR* with early contact-dependent inhibition (96, 97). *FOXP3 *is the most commonly used marker for identifying Treg cells and is an essential transcription factor for the development and maintenance of suppressive function in Treg cells (26). There is a differential methylation region (DMR) upstream of the *Foxp3* promoter in RA Treg cells, with enhancer activity sensitive to methylation-induced silencing. Both the expression of DMR and DNMT1/3 in RA Treg cells were downregulated, and the methylation of DMR was negatively correlated with the mRNA expression of *Foxp3*. RF-negative and-positive Treg cells also express *Foxp3* differently. RF-positive Treg cells express lower *Foxp3* levels and have a higher DMR methylation status (98). Tumor necrosis factor receptor 2 (TNFR2) is an important molecule that regulates *Foxp3* expression. It can inhibit DNA methylation of *the Foxp3* promoter by maintaining its expression in Treg cells. The lack of TNFR2 leads to the deterioration of experimental animal arthritis models, accompanied by a decrease in the number and a defect in the suppressive function of Treg cells. It leads Treg cells to inflammatory phenotypic differentiation of Th17 cells, which attempts to suppress inflammatory cells but fails (99). Consistent with this, the number of a subset of Treg cells expanded during the active stage of RA, similar to the pathogenic T cell phenotype (100). Similarly, the degree of methylation of *Foxp3* in Treg cells of TNFR2 knockout mice increased. Under *in vitro* culture conditions, TNF upregulates *Foxp3* expression in Treg cells through TNFR2 signaling, and TNFR2+ Treg cells increase in patients with RA receiving anti-TNF-α therapy for three months (101). Regular expression of *CD83, SMAD7*, and *CTLA4* can maintain the functional integrity of Treg cells. Mice lacking *CD83* cause abnormal Treg cell differentiation and promote inflammation (31, 32). The down-regulation of CD83 and SMAD7 may be caused by meQTL-mediated differentially methylated regions in RA (11). SMAD7 is significantly reduced in the synovial tissue of patients with RA, and the TGF-β/SMAD3 signal is enhanced considerably, further intensifying the pro-inflammatory response of Th1 and Th17 cells (102). Experimental arthritis mice lacking *SMAD7* developed severe arthritis, including joint swelling, synovial hyperplasia, bone destruction, and immune cell infiltration. The mechanism may be related to the imbalance of inhibitory function in Treg cells, the inflammatory response of Th1 and Th17 cells, and the overactivation of the TGF-β/SMAD3/IL-6 pro-inflammatory signaling pathway (102). In addition, the Treg inhibitory function of patients with RA is impaired, and the expression of *CTLA4* is downregulated, which may be due to methylation of the *CTLA4* promoter. It causes Treg cells to fail to induce the expression of tryptophan-degrading enzyme indoleamine 2,3 -dioxygenase (IDO) and the activation of the immunomodulatory kynurenine pathway (103). Decitabine, a DNA methylation inhibitor, can induce the expansion of RA Treg cells through an IDO-dependent pathway and promote the apoptosis of Th1 and Th17 cells to improve RA (104). DNA methylation may affect Treg cell function, further influencing autoimmune T cells and inflammation in RA.

## The DNA Methylation of T Cells as a Potential Biomarker for Rheumatoid Arthritis

The identification of DNA methylation in RA holds promise for augmenting and improving the clinical diagnosis process. At present, biomarkers for the diagnosis of RA include anti-citrullinated protein antibody (ACPA) and RF. The sensitivity is insufficient, and new markers are still needed to assist ACPA and RF, enabling more accurate diagnoses (105). The addition of other molecular tests to the markers of seropositivity offers an opportunity to increase the sensitivity of the combined test. Prime candidates for this expansion of the diagnostic utility of RA include genetic markers and differentially methylated regions. There are 1951 differentially methylated CpGs of T lymphocytes in patients with early RA, 60% of which were hypermethylated, representing 1216 genes. Of the top 15 genes, *C6orf47, AGPAT9, DUSP22, SLC25A13*, and *SNORA15* were hypermethylated. *MAGI2, C11orf58, POMZP3, ZNF708*, and *CLEC2L* were hypomethylated (28). Similarly, the study identified 509 differentially methylated CpGs of T lymphocytes in RA. The top 15 with gene hypermethylation included *DUSP22, C1orf106, ZFP57, ARSB, LSM5*, and *HIST1H4D*, and hypomethylation included *TMEM9, CCS, FBRSL1, GALNT9, PDHB, MGMT, DDO, KLF6*, and *RDH12* (29). *DUSP22 *has been shown many times to be DNA hypermethylated, which is closely related to the regulation of immunity and inflammation. Its reduced expression can promote CD4+ T cells to differentiate into Th1 and Th17 cells, thereby promoting inflammation (106). Similarly, the inhibition of DUSP22 promotes the activation, proliferation, and differentiation of CD4+T cells into Th1/Th17 cells, thus promoting an inflammatory response (107). In addition, serum DUSP22 levels were negatively correlated with ESR, CRP, and DAS28, and increased in patients with RA receiving DMARDs treatment (108). These results suggest that DUSP22 could potentially be used as a biomarker of RA. In addition, the detection of DNA methylation of the whole genome of CD4+ T cells revealed that the changes in the methylation levels of many genes are related to different aspects of RA. For example, *OR5A2* (cg02981094), *ALDH9A1* (cg03984859), and *C5orf32* (cg02070114) are related to the course of RA. *ZC3H11A* (cg02337583) is related to RF. *OAS2* (cg00085448) was related to the patient’s global assessment. *C16orf71* (cg04705084) and *LOC100129716* (cg00598143) were correlated with DAS28. *SLC38A8* (cg01740650), *C18orf19* (cg00448482), *COL18A1* (cg04760448), *BAT3* (cg05649229), and *PLD3*(cg07071106) were associated with erythrocyte sedimentation rate. *HSPA12A* (cg06942850) is associated with TJC (18). Further experiments are required to verify the accuracy of implementing these interesting results.

## The Pharmacological Mechanism of Drugs for Rheumatoid Arthritis Involves the Regulation of DNA Methylation

Methotrexate (MTX) is a first-line drug for RA treatment. The recommended dose was 25 mg/week. It can be combined with glucocorticoids to achieve better clinical remission (109). DNA methylation analysis of CD4+ T cells purified from the peripheral blood of juvenile idiopathic arthritis revealed 145 differentially methylated sites. When removing four patients receiving MTX treatment, only 11 differentially methylated sites remained, strongly suggesting that the regulatory mechanism of MTX includes regulation of DNA methylation of CD4+ T cells (110). Studies have shown that MTX can affect one-carbon metabolism during the methyl transfer process of CpG island methylation through antifolate metabolism. One-carbon metabolism requires the participation of folic acid (111).. Through further gene pathway analysis of 11 differentially methylated sites, most of them are in the “cellular growth and proliferation, hematological system development and function, hematopoiesis” network centered on TNF-α (110). The study also found that methylated *IL-32* and *MRPL28 *are attractive potential targets (110). The pro-inflammatory factor TNF-α induces the expression of IL-32, which together constitute a chronic autoinflammatory cycle. Anti-TNF-α treatment can significantly reduce IL-32 protein levels in the synovial membrane of patients with RA (45). *MRPL28* is mainly involved in encoding the mitochondrial ribosomal protein L28, and mitochondrial dysfunction may cause CD4+T cells to be sensitive to multiple forms of cell death. Mitochondria are also involved in the energy metabolism of cells, suggesting that the methylation of CD4+ T cells can affect RA in part by regulating the function of mitochondria, which may be a potential mechanism for MTX treatment (46, 47). Significantly, MTX may affect gene expression by regulating the DNA methylation levels of multiple genes in T cells and further regulating multiple immune mechanisms to exert pharmacological effects. T cells in patients with RA who have just started receiving disease-modifying antirheumatic drug (DMARD) treatment show an overall DNA hypomethylation pattern. The expression of DNA methyltransferase 1 (DNMT1) is low, the expression of demethylases (ten-eleven translocation (TET)1, TET2, and TET3) is increased, and the expression of growth arrest and DNA-damage-inducible protein 45A (GADD45A) was downregulated. After MTX treatment, the overall DNA methylation level and DNMT1 levels increased (112). By comparing the methylation levels of whole blood samples of patients with RA who had a good or bad response to MTX therapy for weeks, it was found that the differential methylation sites related to MTX therapy were mainly near *GATA3* (cg27427581), *RPH3AL*(cg21040096),* *and *WDR27*(cg09894276) (52). As mentioned above, *GATA3* mainly promotes the differentiation of Th2 cells, indicating that MTX may partially regulate the methylation and gene expression of GATA3 to regulate the differentiation of CD4+ T cells to treat RA. Interestingly, the whole longitudinal genome of Japanese patients with RA receiving anti-TNF-α therapy showed that the gene expression of *WDR27, MAP3K7, BACH2*, and *GFRA1* might be related to the therapeutic effect, further implying a correlation between DNA methylation, gene expression regulation, and various clinical therapies (53). As mentioned earlier, *ALB* in CD4+ T cells of patients with RA is hypomethylated (54). ALB is involved in the molecular mechanism of MTX treatment in RA (113). In addition, after receiving MTX treatment, the inhibitory function of RA Treg cells was restored, and the mechanism involved increased *Foxp3* and *CTLA-4* expression and reduced the methylation of the upstream enhancer of *Foxp3* (27).

Biological agents are another treatment option for patients with RA who have a poor response to MTX treatment. TNF-α inhibitors are the most widely used. Various biological agents may exert pharmacological effects by regulating DNA methylation (109). By studying the differential gene expression and differential methylation of PBMCs, monocytes, and CD4+ T cells in patients with RA receiving adalimumab (ADA) or etanercept (ETN) before and after treatment, it was found that more than 100 differential genes had DNA methylation changes. Some CD4+T cells of patients with RA who respond to ADA therapy have the characteristics of upregulation of TNF signaling pathway-related genes, such as *CTLA4, TNFSF13B, TNFRSF1B, TNFSF4, IRF1*, and *IL18R1*. In CD4+ T cells of patients with RA responding to ETN therapy, Foxo signaling pathways, such as *FOXO3, FOXO4, TGFBR1*, and *USP7*, were upregulated. NOD-like receptor signaling pathways, such as *GSDMD, RIPK3*, *CASP4*, and JAK/STAT signaling pathways, such as *CISH, SOCS2*, and *PIM1*, were downregulated (114). In addition to anti-TNF-α therapy, the DNA methylation changes of *IL-6* significantly affect the gene expression of early RA, which can promote inflammation through the JAK1/STAT3 pathway (87). The clearance of apoptotic cells by phagocytes has an immunosuppressive effect of preventing the induction of autoimmunity and inflammation. If it cannot be eliminated effectively, it may be harmful. The DNA of apoptotic T cells is demethylated, which may further promote inflammation and stimulate the production of pro-inflammatory factor IL-6 in macrophages by interacting with TLR in RA (50). This further suggests that the existing IL-6R therapy and JAK inhibitor therapy should be applied as soon as possible, and the mechanism may involve DNA methylation in RA. Some drugs or natural components could regulate DNA methylation or hydroxymethylation in diseases like cancer (115, 116). Furthermore, while some of these may have a therapeutic effect in RA, they may not be active directly in treating RA by modulating the DNA methylation status. We still believe some drugs or natural components have therapeutic potential for RA, given some association outcomes. **Table 2** shows updates and summaries thereof. (See **Table 2**)

**Table 2:** Drugs or natural components that regulate DNA methylation or hydroxymethylation.

Name	Effect to DNA methylation or hydroxymethylation	Related disease	Ref.
Azacitidine	Inhibition of DNA methylation	RA	(117)
Decitabine	Inhibition of DNA methylation	RA	(91)
Epigallocatechin-3-gallate	Inhibition of DNA methylation	RA	(118–121)
Epicatechin	Inhibition of DNA methylation	RA	(122, 123)
Caffeic acid	Inhibition of DNA methylation	RA, breast cancer	(124, 125)
Chlorogenic acid	Inhibition of DNA methylation	RA, breast cancer	(125, 126)
Ellagic acid	Inhibition of DNA methylation	RA, breast cancer	(127, 128)
Resveratrol	Inhibition of DNA methylation	RA, breast cancer	(129, 130)
Rosmarinic acid	Inhibition of DNA methylation	RA, breast cancer	(128, 131)
Lsothiocyanates (SFN)	Inhibition of DNA methylation	RA, breast cancer	(132, 133)
Lycopene	Inhibition of DNA methylation	RA, breast cancer	(134, 135)
Parthenolide	Inhibition of DNA methylation	RA, leukemia	(136, 137)
Curcumin	Inhibition of DNA methylation	RA, leukemia	(138, 139)
Procaine	Inhibition of DNA methylation	RA, SS syndrome	(140, 141)
Procainamide	Inhibition of DNA methylation	RA, systemic lupus erythematosus	(142)
Hydralazine	Inhibition of DNA methylation	RA, systemic lupus erythematosus	(142)
Phenethylisothiocyanate	Inhibition of DNA methylation	RA, prostate cancer	(132, 143)
Genistein	Inhibition of DNA methylation	RA, oral cancer	(144, 145)
Quercetin	Inhibition of DNA methylation	RA, colon carcinoma	(146, 147)
Zebularine	Inhibition of DNA methylation	breast adenocarcinoma, multiple myeloma	(148, 149)
RG108	Inhibition of DNA methylation	hearing loss	(150)
Apple polyphenols	Inhibition of DNA methylation	colorectal cancer	(151)
SGI-1027	Inhibition of DNA methylation	colon cancer	(152)
4-amino-N-benzamide analogues	Inhibition of DNA methylation	leukemia	(153)
Cyclophosphamide	Inhibition of DNA methylation	retinoblastoma	(154)
Mahanine	Inhibition of DNA methylation	prostate cancer	(155)
Coffee polyphenols	Inhibition of DNA methylation	breast cancer	(125)
Budesonide	Activation of DNA methylation	lung cancer	(156)
AGI-5198	Inhibition of DNA hydroxymethylation	glioma	(157)
HMS-101	Inhibition of DNA hydroxymethylation	glioma, acute myeloid leukemia, melanoma, thyroid cancer, and chondrosarcoma	(158)

## Conclusion

**Figure 2:**
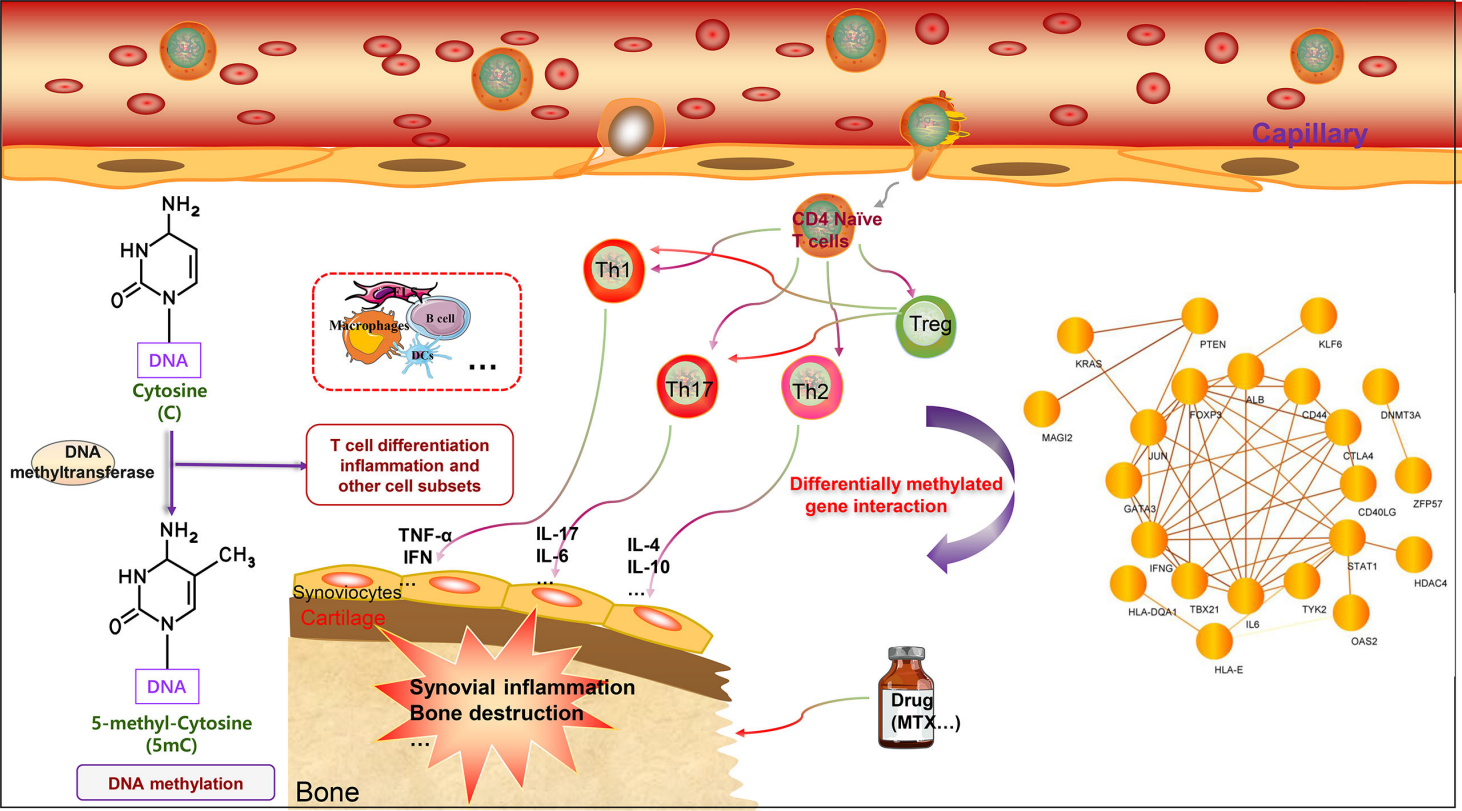
DNA methylation of T cell-related genes affects rheumatoid arthritis. The main characteristics of RA include chronic synovial inflammation and bone destruction. T cells can differentiate into different cell subgroups according to the intracellular microenvironment, including Treg cells, Th1 cells, Th2 cells, and Th17 cells. Th1 and Th17 cells can promote inflammation by secreting different pro-inflammatory factors, including TNF-α, IL-1, IL-6, IL-17, and IFN-γ. Th2 cells mainly secrete cytokines that inhibit inflammation, including IL-4 and IL-10. Treg cells specifically regulate the function, inhibit the differentiation of Th1 and Th17 cells, and thus inhibit inflammation. T cell-related genes can undergo DNA methylation modification to affect gene expression, further regulate T cell function, and affect the progression of RA. DNA methylation genes can also potentially serve as biomarkers for RA and predict drug efficacy. Interestingly, the DNA methylated genes related to T cells can form a network, and there are interactions worthy of further study. In addition, the methylation patterns of multiple immune cells present in RA warrant further investigation.

There are several unresolved issues regarding the role of DNA methylation in RA. First, a cell-specific investigation of the link between DNA methylation and RA is required to fully understand the impact of differential methylation on RA pathophysiology. Many studies have focused solely on the DNA methylation properties of the total cell population, ignoring the variability of cells within distinct cell subsets. There may be several mechanisms by which DNA methylation yields effects, and these effects may be substantially different across cell types. As a result, cell purification is required to properly evaluate DNA methylation and its link with gene expression and regulation of biological functions. There are some shortcomings, including the fact that there is little direct research-based evidence about other T cell subsets (CD8+ T cells, gamma delta T cells, and MAIT T cells) related to DNA methylation regulation. It is undeniable that T cells are heterogenous cell populations. Studying and clarifying the correlation between the DNA methylation patterns of other T cell subsets and RA is very important, and is a potential future research direction. Second, whether DNA methylation begins before the commencement of the disease or varies continually over the course of the ailment requires more well-designed clinical studies for sample collection to clarify its function. The detection of DNA methylation before illness onset can assist in identifying high-risk patients, allowing us to realize the objective of precision medicine. Finally, a distinction should be made between cell-specific alterations in DNA methylation in early and late RA. When comparing various study findings, we should keep the distinctions between chips and detecting systems in mind. Additionally, we mapped the protein interaction network based on the genes that undergo DNA methylation in T cells discussed in this article. Some of the proteins have interactions, potentially focusing on future research for further exploration (see **Figure 2**). In conclusion, integrating cell-specific DNA methylation data with other data, such as primary clinical data, gene expression data, proteomics data, and metabolomics data, would enable us to deliver a more powerful RA-related clinical treatment choice in the future.

## Author Contributions

JZ is responsible for the collection, collation and writing of the original manuscript. KW, CC, LX and PJ is responsible for the collection of the original manuscript. SG, SS, and DH are responsible for the revision and review of the manuscript. All authors reviewed and accepted with the final version.

## Funding

This work was funded by the National Natural Science Funds of China (82074234), Shanghai Chinese Medicine Development Office,National Administration of Traditional Chinese Medicine,Regional Chinese Medicine (Specialist) Diagnosis and Treatment Center Construction Project-Rheumatology,State Administration of Traditional Chinese Medicine, National TCM Evidence-Based Medicine Research and Construction Project, Basic TCM Evidence-Based Capacity Development Program, Shanghai Municipal Health Commission, East China Region based Chinese and Western Medicine Joint Disease Specialist Alliance.

## Conflict of Interest

The authors declare that the research was conducted in the absence of any commercial or financial relationships that could be construed as a potential conflict of interest.

## Publisher’s Note

All claims expressed in this article are solely those of the authors and do not necessarily represent those of their affiliated organizations, or those of the publisher, the editors and the reviewers. Any product that may be evaluated in this article, or claim that may be made by its manufacturer, is not guaranteed or endorsed by the publisher.

## Abbreviations

RA, rheumatoid arthritis; CpG, cytosine guanine (CG) dinucleotide; HLA, human leukocyte antigen; hTERT, human telomerase reverse transcriptase; DVPs, differentially variable positions; KEGG, the Kyoto Encyclopedia of Genes and Genomes; GO, gene ontology; MAPK, the mitogen-activated protein kinase; HBV, hepatitis B virtus; RF, rheumatoid factor; DMP, differentially methylated positions; anti-CCP, cyclic citrullinated peptides; DAS28, disease activity score in 28 joints; CRP, C-reactive protein; SNPs, single nucleotide polymorphisms; NSAID, non-steroidal anti-inflammatory drug; PBMCs, peripheral blood mononuclear cells; IL, interleukin; IFN-γ, interferon-gamma; VDR, vitamin D receptor; FLS, fibroblast-like synoviocyte; TNF-a, tumor necrosis factor-a; RANKL, receptor activator of nuclear factor (NF)−κB ligand; Tfh, T-follicular helper; DNMT, DNA methyltransferase; TNFR2, tumor necrosis factor receptor 2; IDO, indoleamine 2,3 –dioxygenase; MTX, methotrexate; DMARDs, disease-modifying antirheumatic drugs; DNMT1, DNA methyltrasnferase 1; TET1, ten-eleven translocation 1; GADD45A, DNA-damage-inducible protein 45A; ADA, adalimumab; ETN, etanercept; ACPA, anti-citrullinated protein antibody.
